# RDb_2_C2: an improved method to identify the residue-residue pairing in β strands

**DOI:** 10.1186/s12859-020-3476-z

**Published:** 2020-04-03

**Authors:** Di Shao, Wenzhi Mao, Yaoguang Xing, Haipeng Gong

**Affiliations:** 1 MOE Key Laboratory of Bioinformatics, School of Life Sciences, Tsinghua University, Beijing, 100084 China; 2 Beijing Advanced Innovation Center for Structural Biology, Tsinghua University, Beijing, 100084 China

**Keywords:** Mainly β proteins, β-β residue pairing, Protein structure prediction, Ridge detection, Residual neural network

## Abstract

**Background:**

Despite the great advance of protein structure prediction, accurate prediction of the structures of mainly β proteins is still highly challenging, but could be assisted by the knowledge of residue-residue pairing in β strands. Previously, we proposed a ridge-detection-based algorithm RDb_2_C that adopted a multi-stage random forest framework to predict the β-β pairing given the amino acid sequence of a protein.

**Results:**

In this work, we developed a second version of this algorithm, RDb_2_C2, by employing the residual neural network to further enhance the prediction accuracy. In the benchmark test, this new algorithm improves the F1-score by > 10 percentage points, reaching impressively high values of ~ 72% and ~ 73% in the BetaSheet916 and BetaSheet1452 sets, respectively.

**Conclusion:**

Our new method promotes the prediction accuracy of β-β pairing to a new level and the prediction results could better assist the structure modeling of mainly β proteins. We prepared an online server of RDb_2_C2 at http://structpred.life.tsinghua.edu.cn/rdb2c2.html.

## Background

The atomic structures of proteins are fundamental to their functions, and therefore protein structure prediction, the field of computationally predicting the atomic structure of a protein from the amino acid sequence, is always of great importance in protein science. In the last decade, the accuracy of protein structure prediction has been tremendously improved, particularly with the rapid algorithm development in the protein residue contact prediction [1, 2]. Conventionally, two residues are defined as in contact when their C_β_ atoms are positioned within a distance cutoff of 8 Å. Contact information between all residues pairs thus composes a residue contact map, which may provide sufficient distance restraints to improve conformational sampling and model selection or even to directly construct the atomic structure model [3]. The contact map of a protein could be obtained from the multiple sequence alignment (MSA) [4–7], by analyzing the correlated mutations between all pairs of residues in evolution using programs like PSICOV [8, 9], GREMLIN [9], CCMpred [10], FreeContact [11] and PconsC2 [12]. More recently, with the application of computer vision and deep learning techniques in contact prediction, protein residue contacts could be more reliably predicted, for instance, by methods like RaptorX-Contact [13–15], TripletRes [16], DeepMetaPSICOV [17], SPOT-Contact [18] and DeepConPred2 [19], which enormously benefits the tertiary structure prediction of proteins [20].

Despite these advances, structure prediction of the mainly β proteins are still highly challenging. Particularly, the pairing residues in interacting β strands are usually distantly positioned in the amino acid sequence, which toughens the prediction of interacting patterns between β strands and thus the correct identification of topology. The prediction of β-β residue pairing has attracted much attention since 1990s, and many programs have been developed, such as BetaPro [21], MLN/MLN-2S [22, 23], CMM [23] and BCov [20]. These methods, however, rely on the knowledge of native secondary structures during modeling and suffer great performance loss when predicted secondary structures are used.

With the quick development in protein residue contact prediction, β-β residue pairing could be more reliably identified from the predicted residue contact map, because a pair of parallel/antiparallel β strands should exhibit strong contiguous signals in the diagonal/off-diagonal directions even in the presence of noises. As the first β-β contact prediction algorithm that exhibits robust performance in the absence of native secondary structures, bbcontacts uses two hidden Markov models to identify the parallel and antiparallel contacting patterns and achieves a remarkable promotion on prediction accuracy against all previous tools [24]. RDb_2_C, later developed by us, adopts the ridge detection to locate the strong signals of interacting β strands on a predicted contact map and then utilizes a multi-stage random forest framework to refine the β-β residue pairing [25]. Besides the performance gain over bbcontacts, the prediction results of RDb_2_C could further improve the structure modeling of mainly β proteins in practice. Albeit successful, bbcontacts and RDb_2_C are both developed based on the shallow learning techniques, unlike the wide application of deep learning techniques in residue contact prediction.

In this work, we present a second version of RDb_2_C. The new algorithm RDb_2_C2 still uses the ridge detection method to infer the characteristics of interacting β strands [26–29], but engages the residual neural network (ResNet) to further improve the prediction of β-β residue pairing [30]. When compared to the previous version, RDb_2_C2 exhibits a significant improvement (> 10 percentage points) in F1-score in the BetaSheet916 [21] and BetaSheet1452 [20] test sets, and could better facilitate the structure modeling of mainly β proteins.

## Implementation

**Fig. 1:**
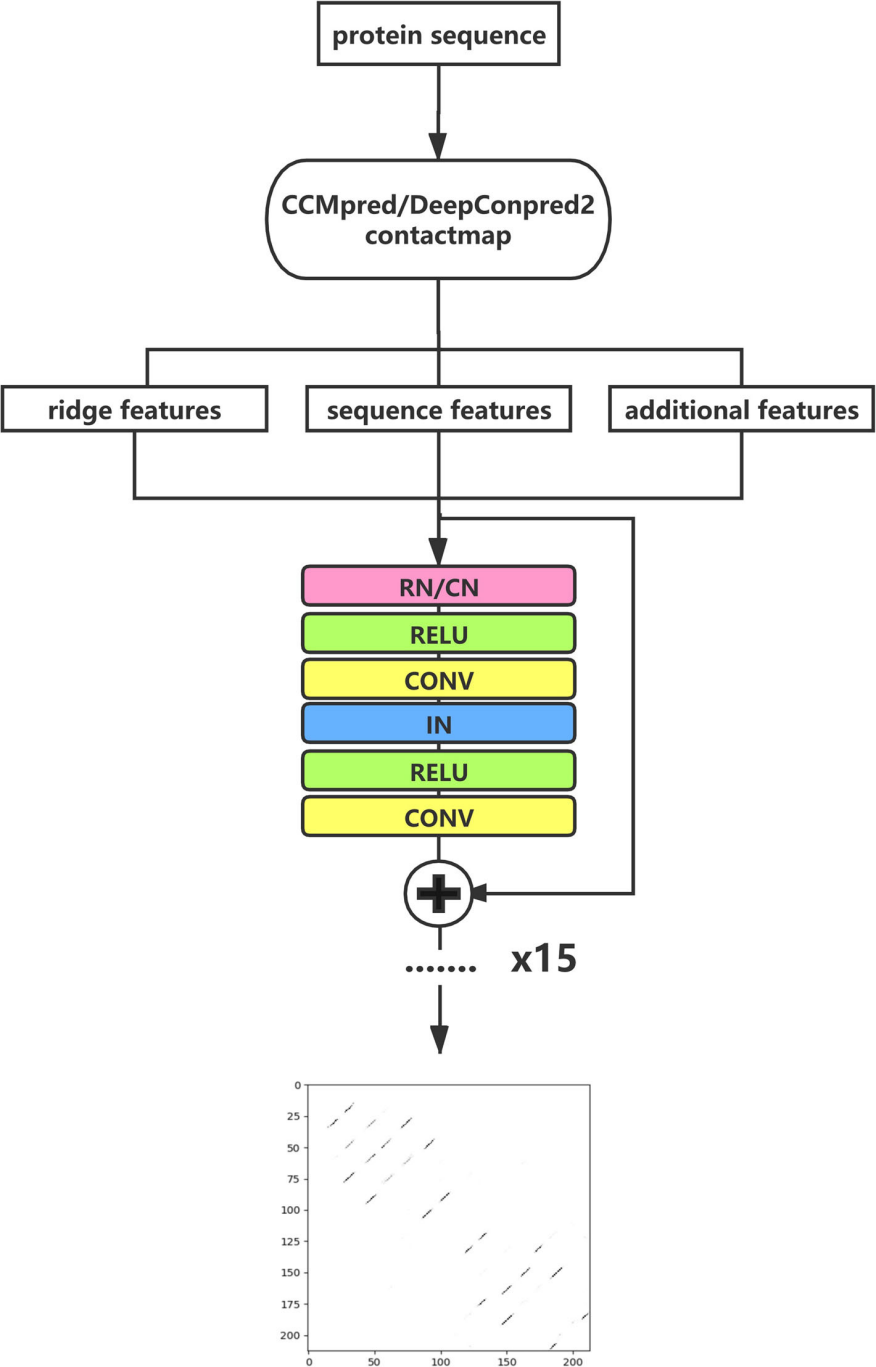
The workflow of our method. RN, CN and IN denote row, column and instance normalizations, respectively. RELU refers to the ReLU activation function. CONV stands for convolution layer

As shown in Fig. 1, for each query protein sequence, RDb_2_C2 starts with the two contact maps predicted from DeepConPred2 and CCMpred, respectively. Similar to the previous version, the algorithm adopts the γ-normalized ridge detection method introduced by Lindeberg to extract the ridge features and also collects sequence features as well as additional features to compose the whole feature set. All features are fed into a ResNet model with 15 blocks for predicting the β-β residue pairing. Notably, in addition to the traditional convolution layers, ReLU activation, instance normalization (IN) and shortcut connection, we also incorporated two normalization operations that have been proved as useful for contact prediction, the row normalization (RN) and column normalization (CN) [31], into the cell-based ResNet structure. Output of RDb_2_C2 is a probability matrix listing the probabilities of all residue pairs to form hydrogen-bonded interactions in β strands.

### Dataset

We established our training set from the protein domain database CATH (version 4.2) [32]. Since RDb_2_C2 focused on residue pairing in β strands, we only retained the domains of the α/β and β categories but removed the overly short ones (< 30 residues). We then eliminated the redundancy within the training set by only retaining the domains in the CATH S35 set (a CATH subset with pairwise sequence identity < 35%) [33]. We took BetaSheet916 [21] and BetaSheet1452 [20], two conventional sets for evaluating β-β contact prediction, as our test sets. Redundancy between the training and test sets were strictly eliminated by removing all domains from the training set that fall into the same CATH fold groups as domains in the test sets. Because the secondary structure prediction method we used (Spider3 [34], see below) could not process the unknown residue X, we deleted all proteins containing residue X in their amino acid sequences. Finally, our training set contained 458 domains, whereas the BetaSheet916 and BetaSheet1452 test sets contained 858 and 1294 domains, respectively.

### Model features and network architecture

RDb_2_C2 adopted the ridge detection method to capture the residue pairing pattern between interacting β strands from the predicted contact maps, as applied in our previous version RDb_2_C. However, we only retained the ridge height and ridge direction as ridge features based on results of feature selection, where the model performance was re-evaluated after removing each type of features. Besides the 2D features like the predicted contact maps and ridge features, we included the following 1D features: secondary structure probabilities predicted by Spider3 and identities of amino acids encoded by one-hot vectors. At last, we took the number of homologous sequences in MSA (following the definition in [13]) and the protein length as 0D features. The 2D, 1D and 0D features were broadcast together as the input for the neural network model. Different from our previous version RDb_2_C, in this work, we adopted Spider3 instead of the DeepCNF [34, 35] to estimate the secondary structure probability, and enriched the raw contact prediction results by DeepConPred2 in addition to CCMpred [10, 19].

We adopted the ResNet architecture in RDb_2_C2 to improve the prediction of β-β residue pairing. Notably, we incorporated two normalization operations that have been proved as useful for contact prediction, RN and CN [31], in the cell-based ResNet structure. Specifically, each ResNet block included two sequential repeats of normalization, leaky ReLU activation and 3 × 3 convolution. However, we applied RN/CN and IN as the normalization operations in the two repeats, respectively (see Fig. 1). We tested architectures with different hyper-parameters: the number of blocks, the number of channels, and whether the RN/CN was applied or not. Starting from 10 blocks and 30 channels without RN/CN, the model performance raised gradually, with the increase of depth and channel number as well as the application of RN/CN. We stopped at 15 blocks and 45 channels with RN/CN applied, in the comprehensive consideration of computational cost and model performance. All models were trained following 5-fold cross validation in the training set, where the cross entropy was taken as the loss function and was optimized by the Adam Optimizer [36] using a learning rate of 1e-4.

### Evaluation

We engaged Precision, Recall and F1-score to measure the algorithm performance. Precision is the fraction of truely predicted instances among all predicted instances, Recall is the fraction of the truely predicted instances among all true instances, and F1-score is the harmonic mean of Precision and Recall:

1 $$ {\displaystyle \begin{array}{l}\mathrm{Precision}=\frac{TP}{TP+ FP}\\ {}\mathrm{Recall}=\frac{TP}{TP+ FN}\\ {}\mathrm{F}1\hbox{-} \mathrm{score}=\frac{2\times \mathrm{Precision}\times \mathrm{Recall}}{\mathrm{Precision}+\mathrm{Recall}}\end{array}} $$

where TP, FP and FN represent true positives, false positives and false negatives, respectively. True samples denote the residue pairs forming β-β hydrogen bonds in the native structure, while positive data denote the residue pairs predicted as forming β-β hydrogen bonds by a predictor. Here, we abandoned the traditional evaluation of the coarse-grained strand-level interaction but focused on the residue-level interaction, because the latter contains more useful information for modeling the 3D structure of a target protein.

### Tertiary structure prediction

Same as our previous work [25], we collected mainly β proteins and generated their tertiary structure models following the CONFOLD protocol by taking the top 1 *L* predictions as distance constraints, where *L* is the protein length. As the native and predicted β-β contacts are always less than 0.5 *L*, these residue pairs are insufficient for reliable modeling. We enriched the residues pairs to 1 *L* by taking the high-ranked and non-redundant contact pairs from the DeepConPred2 results. We adopted distance range of 3.5–6 Å to constrain the C_β_ atoms of residue pairs predicted from RDb_2_C2 that were expected as of high confidence. Simultaneously, we used the distance range of 3.5–10 Å to constrain the C_β_ atoms of residue pairs from DeepConpred2. The best TM-score from the top 5 models was chosen for the evaluation.

## Results

### Model optimization and evaluation

Features and hyper-parameters of our model were optimized based on 5-fold cross validation in the training set, while the model performance was evaluated on two conventional test sets of β-β contact prediction, BetaSheet916 and BetaSheet1452. Table 1 shows the model performance at different numbers of blocks and channels as well as with or without RN/CN operations. Clearly, the model achieves better performances with RN/CN applied and in deeper and wider networks. Finally, in the consideration of both model performance and computational cost, we stopped at the network model of 15 blocks and 45 channels with RN/CN applied.

**Table 1:** F1-scores (%) of models with various hyper-parameters in the 5-fold cross-validation as well as the BetaSheet916 and BetaSheet1452 sets

Evaluation	10 blocks 30 channels w/o RN/CN	10 blocks 45 channels w/o RN/CN	15 blocks 45 channels w/o RN/CN	15 blocks 45 channels w/ RN/CN
Cross-validation	61.82	62.33	62.20	63.17
BetaSheet916	71.60	71.42	71.48	72.08
BetaSheet1452	72.18	72.37	72.08	73.21

**Fig. 2:**
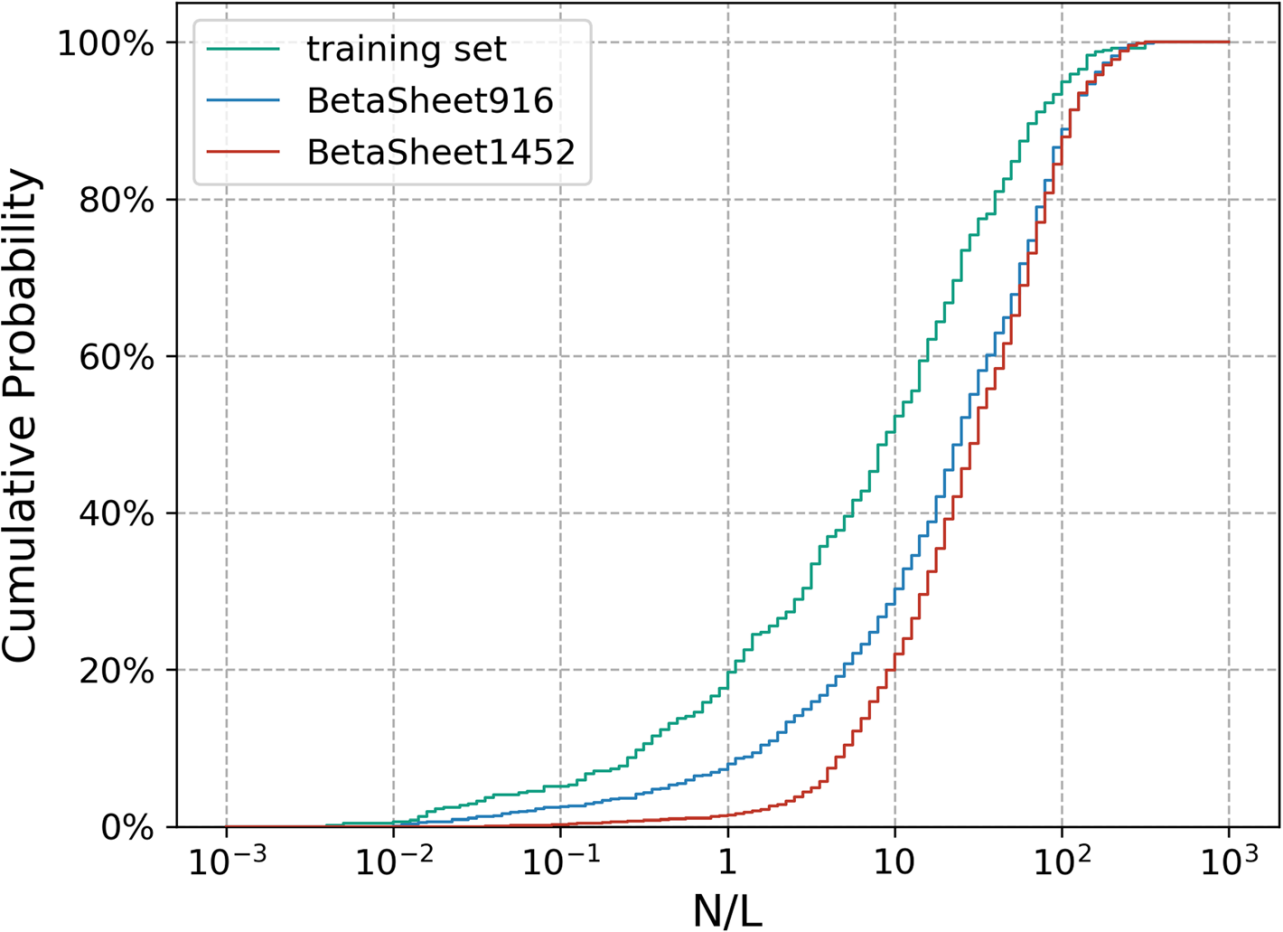
The cumulative distribution of the *N*/*L* in the training set vs. the BetaSheet916 and BetaSheet1452 sets. Here, *N* represents the number of the homologous sequences in the MSA and *L* represents the length of the target protein

The robust performance and steady prediction results of our models in the two test sets support the appropriateness of model training. Interestingly, all tested models show better performance in the test sets than in the cross validation. This is mainly because the proteins in the training/validation set have smaller number of homologous sequences in the MSA and thus are harder targets than those in the test sets (Fig. 2).

Notably, our model was only trained in a small training set of 458 domains, when compared with the BetaSheet916 and BetaSheet1452 test sets that contain 858 and 1294 domains, respectively. In an alternative approach, we enlarged the training set by incorporating all proteins from the BetaSheet916 set, re-trained the model and then tested the performance in the BetaSheet1452 set. The new model only exhibits limited improvement in F1-score (from ~ 73% to ~ 75%). Hence, current choice of training set does not impair the model generalizability significantly.

**Fig. 3:**
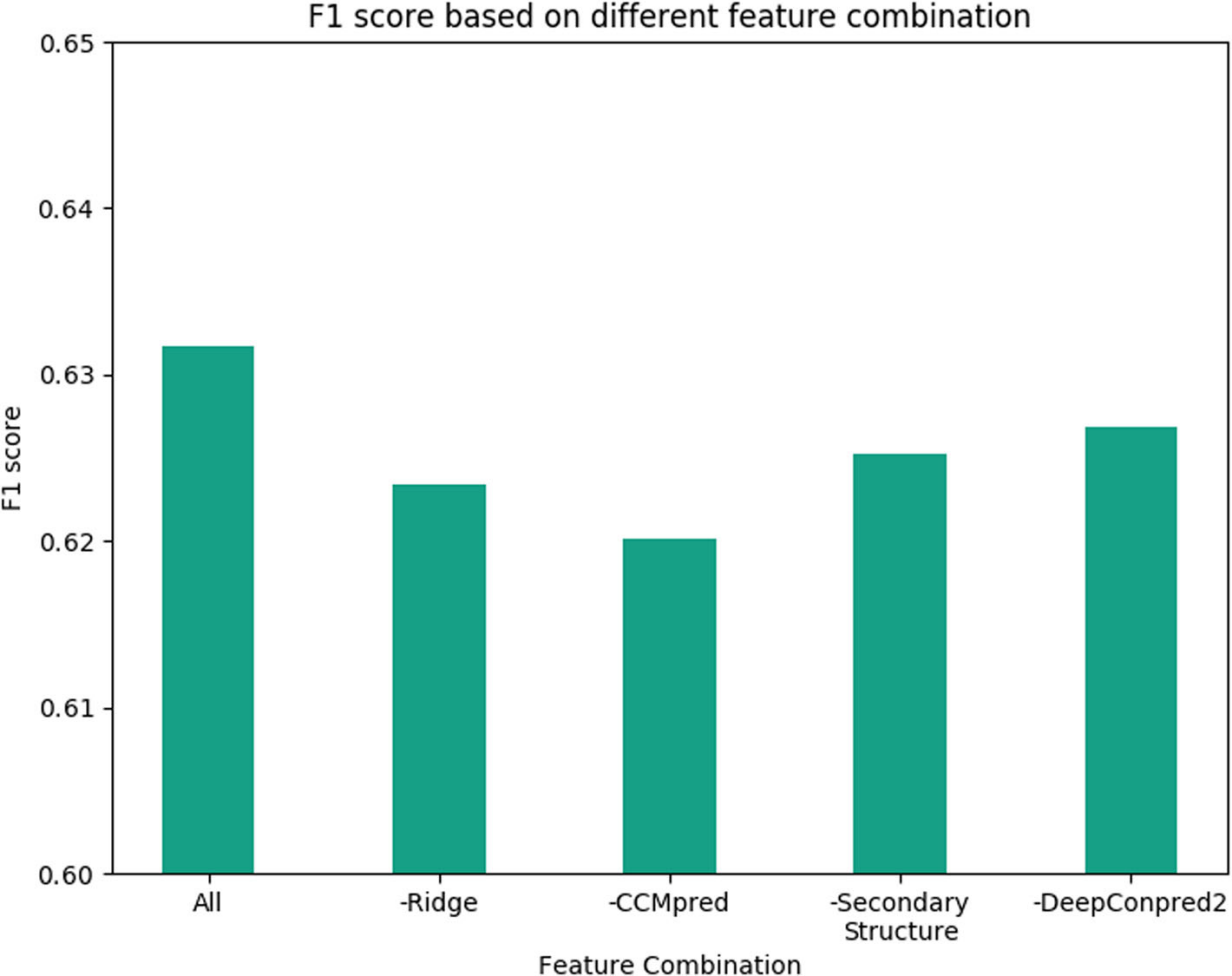
The feature importance to the performance. “-” indicates the corresponding feature was removed in this feature combination

We also evaluated the importance of all features in our final model (15 blocks, 45 channels, with RN/CN) by subtracting the corresponding features and using the new feature combination to re-conduct the model optimization and cross validation. As shown in Fig. 3, all features have positive contribution to the model performance. Particularly, removal of the ridge features elicits a reduction of ~ 1 percentage point to the F1-score, which supports the importance of this feature for extracting β-β pairing information even in the deep-learning-based models.

### Comparison with RDb_2_C and bbcontacts

**Fig. 4:**
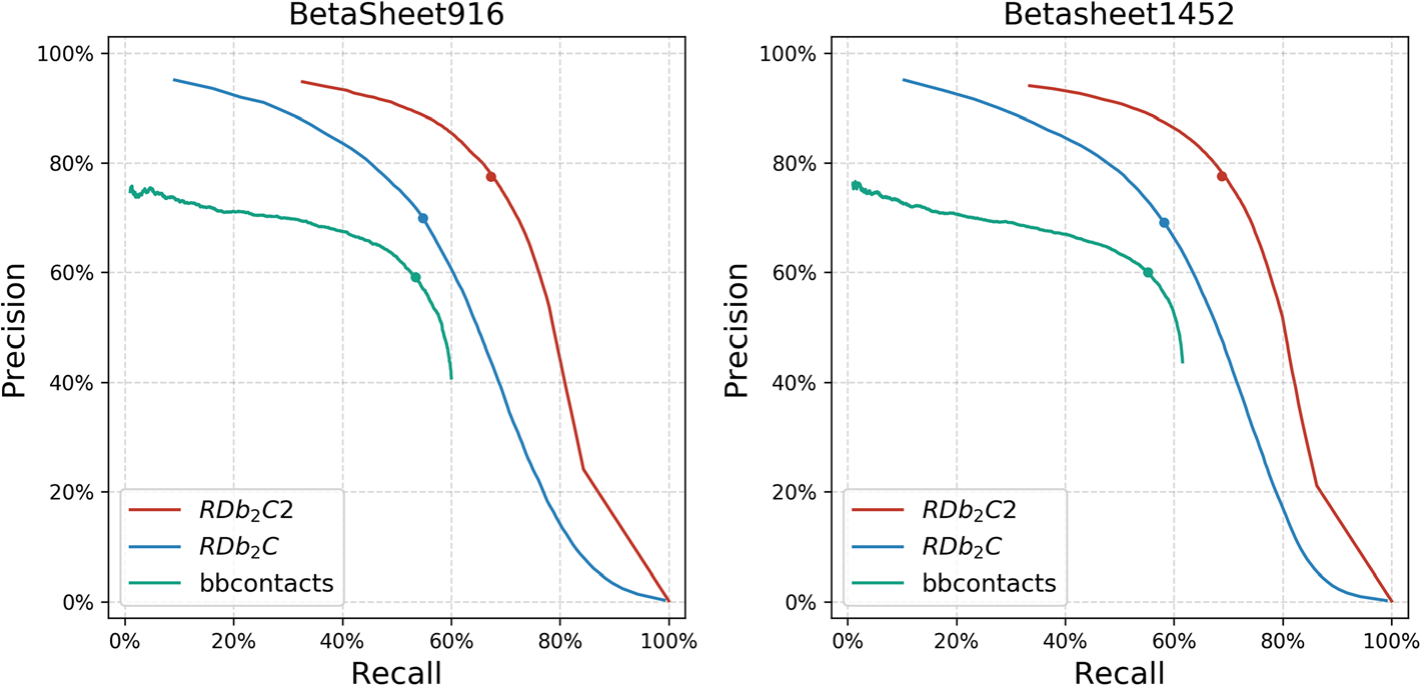
The PR curves of RDb_2_C2 (red), RDb_2_C (blue), bbcontacts (green) in the BetaSheet916 and BetaSheet1452 sets. The dots indicate the suggested cutoff values as optimized in the 5-fold cross validation by F1-scores

We evaluated the performance of RDb_2_C2 against the previous version RDb_2_C as well as another state-of-the-art method bbcontacts in the BetaSheet916 and BetaSheet1452 test sets. Here, the evaluation was conducted at the residue level instead of the strand level, since the detailed pairing information will benefit the structure modeling. As shown by the Precision-Recall (PR) curves, RDb_2_C2 outperforms the other two methods in the whole range by a large margin (Fig. 4). Particularly, at the suggested cutoffs, RDb_2_C2 achieves an F1-score of 72.26 and 73.22% in the BetaSheet916 and BetaSheet1452 sets, respectively. In contrast, the values for RDb_2_C and bbcontacts are 61.45 and 56.15% in the BetaSheet916 set, and 63.18 and 57.52% in the BetaSheet1452 set, respectively. The improvement of RDb_2_C2 over the previous version is > 10 percentage points in F1-scores.

**Fig. 5:**
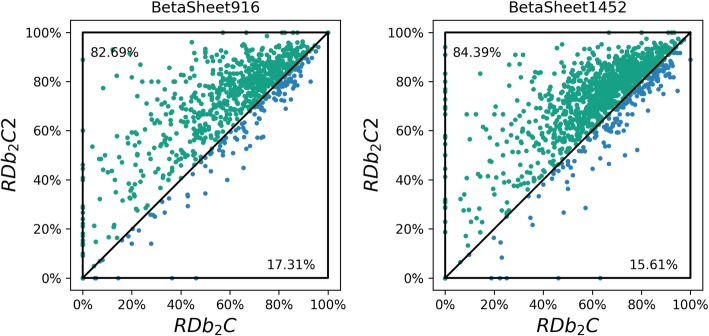
Comparison of RDb_2_C2 and RDb_2_C for individual proteins of the BetaSheet916 and BetaSheet1452 sets. The vertical and horizontal axes represent the F1-scores of RDb_2_C2 and RDb_2_C, respectively. Each dot stands for a protein, with green and blue colors highlighting the cases for which RDb_2_C2 and RDb_2_C win, respectively

We then calculated the F1-scores of RDb_2_C2 and RDb_2_C for individual proteins in two test sets for a more detailed comparison (Fig. 5). Clearly, RDb_2_C2 remarkably outperforms the previous version: 82.69% of the proteins in the BetaSheet916 set have higher F1-scores in the RDb_2_C2 prediction, whereas the number slightly increases to 84.39% in the BetaSheet1452 set.

Protein contact prediction has achieved significant advances in recent years and highly accurate contact maps may intrinsically contain the residue pairing information between β strands. To further validate the necessity for the development of specific β-β residue pairing predictors, we compared our method with a recently developed, end-to-end differentiable contact predictor DeepECA [37] for inferring the β-β residue pairing on BetaSheet916 and BetaSheet1452 sets. Notably, we extracted predicted contacts between β residues (“E” or “B” in the DSSP [38] definition) in the DeepECA prediction results for evaluation, which may slightly overestimate the performance of this program because of the utilization of knowledge of native secondary structure. Table 2 lists the precision, recall, F1-score and AUPRC (i.e. area under the PR curve) values for DeepECA and RDb_2_C2 as well as RDb_2_C and bbcontacts. Clearly, pure contact predictors like DeepECA underperform specifically developed predictors like RDb_2_C2, RDb_2_C and bbcontacts in the prediction of β-β residue pairing. Considering the importance of hydrogen-bonded β-β residue pairing information in the structural modeling of mainly β proteins, methodological development of specific β-β residue pairing prediction is still essential.

**Table 2:** Comparison of RDb_2_C2 against DeepECA, RDb_2_C and bbcontacts on proteins from the BetaSheet916 and BetaSheet1452 sets

		Precision (%)	Recall (%)	F1-score (%)	AUPRC (%)
BetaSheet916	RDb_2_C2	77.34	67.80	72.26	73.29
	DeepECA	21.31	60.76	31.55	16.24
	RDb_2_C	69.91	54.81	61.45	59.88
	bbcontacts	59.18	53.41	56.15	NA
BetaSheet1452	RDb_2_C2	78.71	68.44	73.22	74.15
	DeepECA	20.99	60.24	31.13	15.83
	RDb_2_C	69.10	58.19	63.18	61.87
	bbcontacts	60.04	55.21	57.52	NA

Evaluation of AUPRC is not applicable for bbcontacts, because this program only outputs prediction results for a part of residues pairs with high scores. Precision and recall values are obtained at the cutoff of optimal F1-score

### Contribution in tertiary structure prediction

Accurate prediction of β-β pairing should be capable of assisting the structure modeling of mainly β proteins. In order to evaluate the effectiveness of our method in the tertiary structure prediction, we chose 61 mainly β proteins (i.e. with ≥50% β residues) from the BetaSheet916 set as in our previous work [25], and used the standard CONFOLD protocol to fold these proteins by applying the predicted β-β contacts as constraints [39]. As the native and predicted β-β contacts are always less than 0.5 *L* (*L* is the number of residues in a protein) and are thus insufficient for model constraining, we enriched the contacting residue pairs to 1 *L* by adding the high-ranked and non-redundant pairs from the results of DeepConPred2. Same to our previous work, constraints of 3.5–6 Å were applied to the predicted β-β residue pairs, while constraints of 3.5–10 Å were applied to the enriched pairs. For each target protein, the best TM-score [3] from the top 5 models was chosen for the evaluation.

**Fig. 6:**
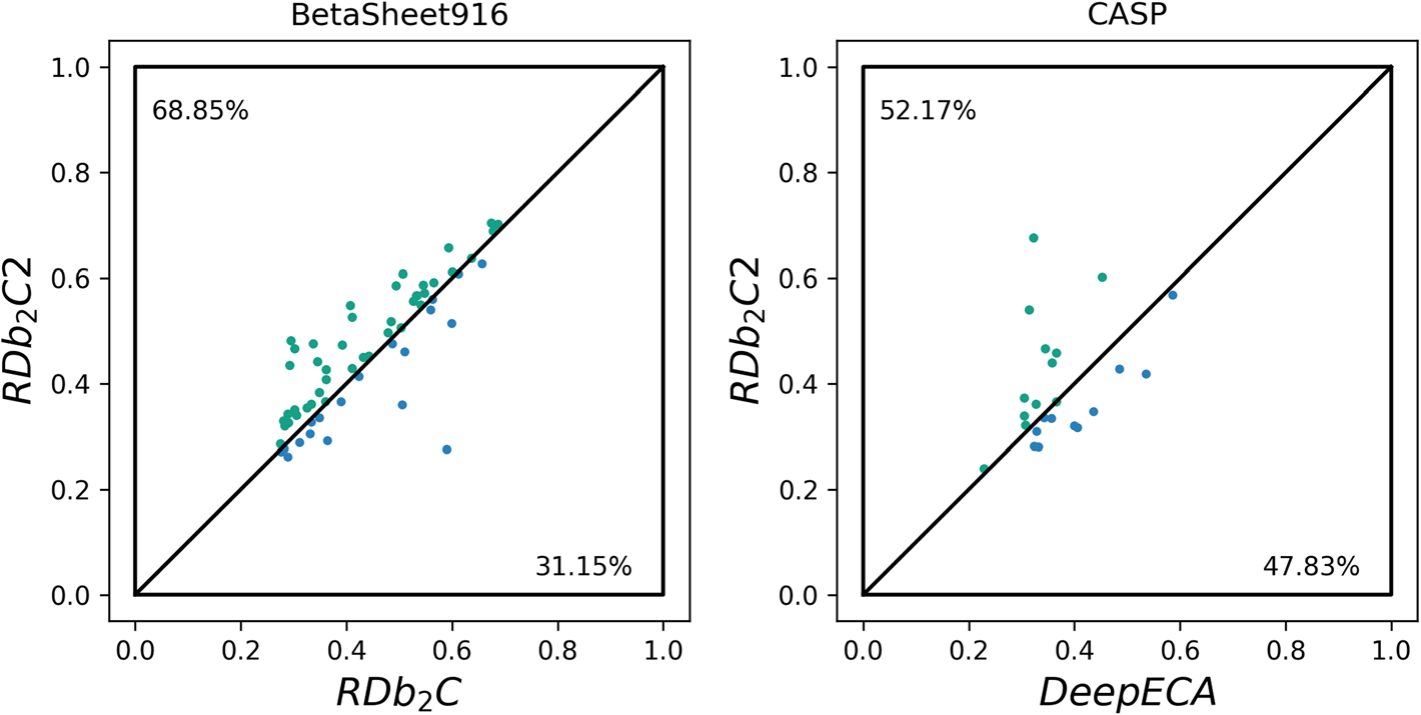
Comparison of TM-scores for structure models constructed using the prediction results of RDb_2_C2 vs. other predictors. In the left panel, RDb_2_C2 is compared with RDb_2_C on mainly β proteins in the BetaSheet916 set. In the right panel, RDb_2_C2 is compared with DeepECA on mainly β proteins collected from CASP11–13 datasets. The green and blue dots represent the target proteins that have better structure models when folded using prediction results of RDb_2_C2 and the rival methods, respectively

As shown in the left panel of Fig. 6, for 68.85% of tested proteins in the BetaSheet916 set, structure models generated by the prediction results of RDb_2_C2 have higher TM-scores than those generated by the previous version. This indicates that the improvement in β-β pairing by RDb_2_C2 indeed enhances the model quality for the tertiary structure prediction.

Subsequently, we collected 23 mainly β proteins (i.e. with ≥50% β residues) from the CASP11–13 datasets (see Table S1) and folded them using the same protocol. In the control experiment, we folded these proteins using the top 1 *L* predicted contacts of DeepECA as constraints (3.5–8 Å for general contacts in CONFOLD). As shown in the right panel of Fig. 6 and also in Table S1, structure models generated using our method achieve better quality, which further supports the essential role of β-β residue pairing prediction algorithms in the tertiary structure prediction of mainly β proteins.

### Running time, memory cost and availability

For a 100-residue protein, the overall time and memory cost for the RDb_2_C2 prediction are 10 min and 9GB, respectively. We prepared an online server of RDb_2_C2 at the website of http://structpred.life.tsinghua.edu.cn/rdb2c2.html.

## Conclusions

We employed the ResNet architecture to produce a new version of our ridge-detection-based β-β pairing predictor. The new algorithm RDb_2_C2 exhibits remarkable improvement over the previous version not only in the prediction accuracy of β-β contacts, but also in the contribution to practical structure modeling for mainly β proteins. Ridge features still make positive contribution in the inference of β-β residue pairing information.

## Supplementary information

**Additional file 1: Table S1.** List of mainly β proteins collected from the CASP11–13 datasets.

## Data Availability

All source codes are available at http://structpred.life.tsinghua.edu.cn/Software.html or https://github.com/DeeShao/RDB2C2. An online server of RDb_2_C2 is also available at http://structpred.life.tsinghua.edu.cn/rdb2c2.html.

## Abbreviations

MSA: Multiple sequence alignment
ResNet: The residual neural network
PR curves: Precision-recall curves
Neg: Negative
Pos: Positive
TP: True positive
FP: False positive
FN: False negative
RN: Row normalization
CN: Column normalization
IN: Instance normalization
