# Supplementary material for: RDb2C2: an improved method to identify the residue-residue pairing in β strands
Source: BMC Bioinformatics. 2020 Apr 3;21:133. doi: 10.1186/s12859-020-3476-z (PMC7126467; doi:10.1186/s12859-020-3476-z)
Supplement: Supplementary file 1 — Additional file 1: Table S1. List of mainly β proteins collected from the CASP11–13 datasets. [file 12859_2020_3476_MOESM1_ESM.pdf]

Table S1. List of mainly β proteins collected from the CASP11-13 datasets.

Targets	Length	β residues (%)	TM-score	
			Our method	DeepECA
T0766-D1	108	54.63	0.540	0.314
T0780-D1	95	54.74	0.568	0.586
T0780-D2	96	51.04	0.602	0.453
T0782-D1	110	60.91	0.458	0.365
T0784-D1	125	61.60	0.366	0.366
T0785-D1	112	59.82	0.281	0.332
T0796-D1	296	60.14	0.239	0.228
T0800-D1	212	63.68	0.373	0.305
T0808-D1	131	68.70	0.335	0.342
T0822-D1	114	53.51	0.348	0.435
T0833-D1	108	70.37	0.467	0.345
T0857-D1	96	52.08	0.310	0.328
T0860-D1	136	59.56	0.335	0.356
T0872-D1	88	51.14	0.339	0.304
T0886-D1	70	52.86	0.321	0.307
T0891-D1	113	62.83	0.419	0.535
T0900-D1	102	60.78	0.282	0.324
T0921-D1	138	53.62	0.320	0.399
T0928-D1	344	53.20	0.361	0.327
T0954-D1	337	54.90	0.676	0.322
T0960-D5	105	60.00	0.428	0.485
T0963-D5	94	54.26	0.317	0.406
T0968s2-D1	115	56.52	0.440	0.358
Average			0.397	0.370
